# Clinical laboratory as an economic model for business performance analysis

**DOI:** 10.3325/cmj.2011.52.513

**Published:** 2011-08

**Authors:** Vikica Buljanović, Hrvoje Patajac, Mladen Petrovečki

**Affiliations:** 1 Specialized Medical Biochemistry Laboratory, Našice General Hospital, Našice, Croatia; 2 Adris group d.d., Rovinj, Croatia; 3 Rijeka University School of Medicine, Rijeka, Croatia; 4 Dubrava University Hospital, Zagreb, Croatia

## Abstract

**Aim:**

To perform SWOT (strengths, weaknesses, opportunities, and threats) analysis of a clinical laboratory as an economic model that may be used to improve business performance of laboratories by removing weaknesses, minimizing threats, and using external opportunities and internal strengths.

**Methods:**

Impact of possible threats to and weaknesses of the Clinical Laboratory at Našice General County Hospital business performance and use of strengths and opportunities to improve operating profit were simulated using models created on the basis of SWOT analysis results. The operating profit as a measure of profitability of the clinical laboratory was defined as total revenue minus total expenses and presented using a profit and loss account. Changes in the input parameters in the profit and loss account for 2008 were determined using opportunities and potential threats, and economic sensitivity analysis was made by using changes in the key parameters. The profit and loss account and economic sensitivity analysis were tools for quantifying the impact of changes in the revenues and expenses on the business operations of clinical laboratory.

**Results:**

Results of simulation models showed that operational profit of €470 723 in 2008 could be reduced to only €21 542 if all possible threats became a reality and current weaknesses remained the same. Also, operational gain could be increased to €535 804 if laboratory strengths and opportunities were utilized. If both the opportunities and threats became a reality, the operational profit would decrease by €384 465.

**Conclusion:**

The operational profit of the clinical laboratory could be significantly reduced if all threats became a reality and the current weaknesses remained the same. The operational profit could be increased by utilizing strengths and opportunities as much as possible. This type of modeling may be used to monitor business operations of any clinical laboratory and improve its financial situation by implementing changes in the next fiscal period.

Increasing health care expenses and implementation of rationalization have brought about the need for a detailed computer modeling of economic business operations on which is based the analysis of health care management (1) and monitoring of spending, timing, and sources of largest or new expenses, and possible savings without changing the quality of the work performed (2).

Although a hospital-based clinical laboratory is an integral part of a health care system, it may be observed as a separate unit. Quality management and good work organization aimed at achieving a continuous improvement in pre-analytical, analytical, and post-analytical phases are part of a good laboratory practice (3-5). Successful quality management in a clinical laboratory setting reduces both the need to repeat tests and raw material consumption, leading to increased business effectiveness.

Analysis of business expenses of a clinical laboratory and calculation of the cost of laboratory tests under different work conditions are part of the laboratory manager’s job (6). For an overall picture of business performance, the manager has to have a detailed knowledge about revenues and expenses (7), which are used in the assessment of the financial profitability of the laboratory in general and individual laboratory tests in particular (8).

Increased productivity of clinical laboratories is directly associated with the technological development of laboratory diagnosis (9) through automation, infomatization, computer networking, consolidation, and integration. Automation is a process in which laboratory analyzers carry out a large number of tests with a minimal participation of laboratory staff (10). Consolidation is a merger of laboratory and different specialties into new, separate units (eg, cytology, pathology, clinical chemistry, transfusiology, nuclear medicine, or microbiology). Integration is association of laboratories at different levels of health care system into an integrated laboratory.

Implementation of laboratory information system may improve work organization, time saving, and control of the work process (11,12). In the beginning, a new technology incurs expenses because it requires financial investment to be made. Financial effectiveness of investments may be predicted by using an economic model, ie, a model of laboratory as an economic unit, and by calculating expected expenses and revenues resulting from the changes in the work of laboratory, which allows making of justifiable decisions based on the measured parameters.

To develop the laboratory model as an economic unit, revenues and expenses have to be compared over a defined period of time, usually, over one year. The difference between total revenues and total expenses clearly shows whether or not the laboratory is profitable and presents a starting point for choosing the right measures to improve laboratory business operations.

The aim of the study was to analyze business operations of a clinical laboratory as an economic unit. For that purpose, we used a SWOT analysis, ie, the analysis of strengths, weaknesses, opportunities, and threats, to identify the possibilities of improving laboratory business operations. Based on the SWOT analysis, it is necessary to identify and present possible calculated models of economic operations for the clinical laboratory. An economic sensitivity analysis of these business models revealed the factors influencing the profitability of clinical laboratory's business operations. We hypothesized that comparison of these models enables decision-making for the economic benefit of a clinical laboratory.

## Material and methods

### SWOT analysis

Business conditions and possibilities to improve business operations of a clinical laboratory were investigated by using the SWOT analysis, which reveals strengths, weaknesses, opportunities, and threats that can have an effect on an organization unit (in the order as presented in Table 1), ie, clinical laboratory (13,14). Strengths and weaknesses refer to internal characteristics of a clinical laboratory, whereas opportunities and threats are external factors. Strengths describe advantages of the laboratory in comparison with other similar laboratories in the same geographical region of 50 km in diameter, covering a population of approximately 100 000. Opportunities refer to conditions in the laboratory that may lead to the improvement in business operations. Weaknesses refer to shortcomings in the work of laboratory and removing the weaknesses would create new opportunities that could improve the quality of work and, consequently, increase profitability. Threats represent conditions that may lead to difficulties in business operations (15).

**Table 1:** SWOT analysis of the Clinical Laboratory at Našice General County Hospital

STRENGHTS	WEAKNESSES
Available to patients 24 h a day	No information system
Wide range of different laboratory tests	Incomplete automation
Profitable performance	High direct labor expenses (gross salaries)
	No system of measuring labor efficiency

OPPORTUNITIES	THREATS
Automation	Reduced tests for outpatient services
Informatization	Reduced tests for primary health care
Reduction in direct labor expenses	New laboratory opening
Additional services currently provided by other laboratories in the region	

The SWOT analysis reveals possibilities for optimization of laboratory work if work-related weaknesses and external threats are reduced or completely removed, strengths of the laboratory increased, and most or all opportunities utilized.

Data for the SWOT analysis were collected from (a) publications (by type and number) affiliated to the laboratory over a single calendar year; (b) expenses of laboratory material (data obtained from the hospital pharmacy); (c) gross salaries for laboratory staff (data obtained from the administrative office); and (d) overhead and common hospital expenses (eg, education of residents, building maintenance, etc.), which make 7% of the total hospital expenses (percentage of the expenses equals the percentage of hospital employees working in the laboratory) and are covered by the laboratory.

Opportunities were analyzed (additional tests performed in the laboratory) by listing all the tests performed within a systematic physical examination, tests required for physician's certificate, and tests that are directly paid and provided by other clinical laboratories in the region in the same time period.

### Profit and loss account

We analyzed business operations of the laboratory in one-year period, using a profit and loss account to express the laboratory's profitability. The basic elements in the profit and loss account included revenues, expenses, and their difference, which expressed final profit or loss and showed the laboratory's profitability. Revenues come from fees charged for the tests performed, and expenses refer to all expenses needed to earn revenues.

To use this method for analysis of laboratory's business performance, for the purpose of profit and loss account, revenues and expenses were additionally divided into different categories. Revenues were divided according to the patient's referral (hospital, outpatient services, primary care) and other sources, such as financial donations or direct financial remuneration for provided laboratory services (profit and loss account, Table 2). Expenses were divided into direct material expenses, other production expenses (direct labor and overhead expenses), and general expenses (indirect and others).

**Table 2:** The profit and loss account for the Clinical Laboratory at Našice General County Hospital in 2008 and models created by economic sensitivity analysis (in €)

Profit and loss account	Real system	Models					
		automation	informatization	three employees less	additional tests	reduced tests for outpatient services	reduced tests for primary health care
**(1) Revenue**							
(1.1) hospital	534 241	534 241	534 241	534 241	534 241	534 241	534 241
(1.2) outpatient services	311 049	311 049	311 049	311 049	311 049	91 991	311 049
(1.3) primary health care	340 740	340 740	340 740	340 740	340 740	340 740	0
(1.4) other	10 899	10 899	10 899	10 899	75 342	10 899	10 899
**Total revenue**	**1 196 929**	**1 196 929**	**1** **196 929**	**1 196 929**	**1 261 372**	**977 871**	**856 189**
**(2) Expenses**							
(2.1) directs materials expenses	211 895	231 983	211 895	231 983	228 879	173 721	139 152
(2.2) other production expenses	366 699	366 699	366 699	310 783	366 699	366 699	366 699
(2.2.1) direct labor	352 128	352 128	352 128	296 212	352 128	352 128	352,128
(2.2.2) production overhead expenses	14 571	14 571	14 571	14 571	14 571	14 571	14 571
(2.3) general expenses	147 912	147 912	166 783	166 783	147 912	147 912	147 912
(2.3.1) indirect	125 925	125 925	126 889	126 889	125 925	125 925	125 925
(2.3.2) other	21 987	21 987	39 894	39 894	21 987	21 987	21 987
**Total expenses**	**726 506**	**746 594**	**745 377**	**709 549**	**743 490**	**688 332**	**653 763**
**(3) Contribution margin (1-2.1)**	**985 034**	**964 946**	**985 034**	**964 946**	**1 032 493**	**804 150**	**717 037**
**(4) Gross profit (3-2.2)**	**618 335**	**598 247**	**618 335**	**654 163**	**665 794**	**437 451**	**350 338**
**(5) Operating profit (4-2.3)**	**470 423**	**450 335**	**451 552**	**487 380**	**517 882**	**289 539**	**202 426**

A profit and loss account provides a profit breakdown expressed through a contribution margin, gross profit, and operating profit. A contribution margin is the total revenue (charged laboratory tests and other income) minus direct material expenses. Gross profit is the difference between the total revenue and total production expenses (direct material expenses and other production expenses). Operating profit with a positive number sign indicates profitable business operations, whereas negative number sign indicates unprofitable business operations.

### Economic sensitivity analysis

Economic sensitivity analysis was used to show how business operations may be changed by changing one or more parameters in the profit and loss account (16). This type of analysis showed the influence of changes in the revenues or expenses on business operations, ie, profitability of the clinical laboratory expressed as operating profit. By changing input parameters, revenues or expenses, we created six models for changing operating profit in comparison with the basic model of clinical laboratory business operations. In other words, economic sensitivity analysis was based on the data obtained by SWOT analysis.

Data for the following models are shown (Table 2): automation, informatization, three employees less, additional tests, reduced tests for outpatients, and reduced test for primary health care.

Data on income in Croatian Kuna (HRK) were converted at the exchange rate of €1 to HRK 7.26 (June 2010 exchange rate list).

### Institution

The Clinical Laboratory at Našice General County Hospital was chosen as a case laboratory for this study. The SWOT analysis and profit and loss account were performed for the fiscal year of 2008, which corresponds to a calendar year. The Clinical Laboratory is a typical provider of clinical laboratory services for all hospital in-patients, hospital outpatients, and patients referred from primary care offices in the region. The Našice General County Hospital is a non-profitable health institution supported by the state budget through the Croatian Institute of Health Insurance.

## Results

### SWOT analysis

Table 1 shows the SWOT analysis results. Strengths include availability to patients 24 hours a day, which is a unique service offered by no other laboratories receiving patients referred from primary practice in the region; wide range of different tests; and profitable business operations in 2008 (Table 2). Weaknesses consist of the lack of information system, incomplete automation, high costs of direct labor, and a lack of system for measuring labor efficiency of the laboratory staff.

Opportunities include automation, informatization, reduction in direct labor expenses along with the implementation of automation and informatization, and increase in additional tests that are currently performed by other laboratories in the region. Threats include possible cessation of tests for outpatient services and patients referred from primary care.

### Clinical laboratory model

The basic model of clinical laboratory as an economic unit shows the actual business operations in 2008 (Table 2, real system). In 2008 fiscal year, the clinical laboratory operated profitably and realized a positive operating profit of €470 423 (Table 2).

### Economic modeling of a clinical laboratory

Economic sensitivity analysis was used to develop six calculated models to change the operating profit with regard to the basic model. The models include automation model, informatization model, “three employees less” model, additional tests model, reduced laboratory tests for outpatient services model, and reduced laboratory tests for primary care model.

The automation model (Table 2) showed that the operating profit decreased to €450 335 in comparison with operating profit produced by the basic model, but increased the expenses related to laboratory reagents (direct material expenses) by €20 088 to cover for the cost of laboratory automation. The informatization model (Table 2) increased other expenses by €18 871, which led to a decreased operating profit of €451 552 in comparison with that produced by the basic model.

By simultaneous informatization and automation, it is possible to reduce direct labor expenses by reducing the number of staff (gross salaries) by three, as shown by the “three employees less” model (Table 2). According to this model, operating profit was €487 380 (three gross salaries amount to €55 916), ie, it increased by €16 957 in comparison with the operating profit of the basic model.

The additional tests model (Table 2) assumed increased number of tests performed as part of systematic physical examination, for the purpose of physician's certificate, and upon personal request by the patient (direct payment), which is a service currently offered by other laboratories in the region. According to this model, the operating profit would increase by €47 459 and amount to €517,882. Other revenue would increase by €64 443 (€75 342-10 899, Table 2) and expenses would increase by €16 984 (€228 879-211 895, Table 2), equaling the cost of laboratory reagents used for additional tests.

The reduced laboratory test for outpatient services model (Table 2) reduced the number of specialized laboratory tests and consequently the revenue in this category. According to this model, the operating profit was reduced by €180 884, totaling €289 539. In the model of reduced laboratory testing for primary care (Table 2), there were no tests performed for primary care patients and, consequently, there was no revenue in this category. The total revenue was, therefore, reduced by the revenue in this category and led to a decrease in the operating profit of €267 997 in comparison with that produced by the basic model, totaling €202 426.

## Discussion

In this study, the SWOT analysis showed strengths, weaknesses, opportunities, and threats for the Clinical Laboratory at Našice General County Hospital. The important strength of the laboratory was its 24-hour availability every day of the year, because the laboratory works around the clock in three shifts. None of the laboratories receiving patients referred from primary care and located within 50 km from the Našice General County Hospital are constantly available, but operate in two shifts on work days only. The next strength was the wider range of tests offered in comparison with the range of tests performed by other laboratories for the primary care, followed by the profitable business operations. The profitable business operations were shown by the profit and loss account, where operating profit in 2008 was €470 423. The strengths of the laboratory should be further developed by including new tests whose profitability should be evaluated beforehand, as required by good financial management (17). New tests should be introduced according to the market demands, which should be investigated through interviews with physicians in the geographic region and health insurance companies.

The weaknesses of the laboratory include lack of informatization and incomplete automation, high expenses of direct labor, ie, gross salaries, which account for 4% of total expenses, and lack of the system for measuring labor efficiency. It is not possible to financially stimulate the laboratory staff to work more efficiently, because the laboratory is an organizational unit within the hospital financed from the state budget and each employee receives a salary determined by the number of points for their professional qualification level and conditions of work. This salary system is used statewide in Croatia and cannot be changed by the laboratory manager. This weakness may be reduced by finding other possible ways to motivate the staff and value their work in a non-financial way, for example, by internal reorganization of the laboratory where one employee would be entrusted with managing 2-3 other laboratory employees and thus earn respect and feel more satisfied. In the following year, automation and informatization could be implemented. Irrespective of the cost, the benefits of informatization and automation are unquestionable as they substantially increase the quality of services (18). Consequently, according to our estimate, informatization and automation would reduce by three the number of employees required to perform the same number of laboratory tests as in the analyzed year, which would then reduce the direct labor expenses, ie, gross salaries expense. Reduction in direct labor expenses would be permanent. The cost of possible automation of €20 088 would be covered by increased price of laboratory reagents over a specified period of time, usually a five-year period. After the automation was paid off, the price of reagents would be reduced back to original one. Informatization that would cost €18 871 could be fully paid off at once.

It should be noted that the investment in informatization is treated as a one-time expense, meaning that the informatization purchase value is expressed as an expense in the year when the purchase was made. This rule applies to all institutions financed by the government budget. If the laboratory was privately owned, the purchase value of the informatization equipment of €18 871 would be divided over four years, increasing the annual expenses over the four-year period by €4 717.75. By automation, informatization, and reduced number of employees, the weakness of having high labor expenses would be transformed into an opportunity to increase laboratory test volume and reduce labor expenses. In this way, interdependence of calculated models is demonstrated because the expenses of automation (€20 088) and informatization (€18 871) are lost by reducing the expenses of the gross salaries of the three employees (€55 916).

New opportunity for every clinical laboratory would be to take on additional laboratory tests. Studies into market expansion show that profitability is increased with an increase in the number of users, which is the usual practice in the United States and other market-oriented countries (19). Additional work for the Clinical Laboratory at Našice General County Hospital would include laboratory tests that are currently not performed at the clinical laboratory but are performed at other laboratories in the close region and include tests for the purpose of issuing physician's certificates, tests performed within systematic physical examinations, and tests paid directly by the patients. By performing additional tests, the operating profit would increase by €47 459. If possibilities and opportunities were realized, the operating profit would increase to €535 804, which unifies simultaneous application of the first four models: automation, informatization, three employees less, and additional tests. Together they would represent the best model to improve business operations.

The threat to the clinical laboratory is the establishment of a new clinical laboratory that would take over part of the patients and thus lead to the reduction in the number of tests performed, as shown by the models of economic operations: models of reduced tests for outpatient services and reduced tests for primary care. If this threat became a reality, the operating profit would be reduced to €202 426 (€267 997 less). Patients can request specialized tests in other laboratories if they are willing to pay travel expenses to a remote laboratory. Reduced tests for outpatient services would lead to a reduction in the operating profit by €180 884, ie, the operating profit would amount to €289 539. If a new laboratory was established, it would substantially reduce the operating profit of the clinical laboratory, because the laboratory could take over patients referred from the primary care and perform specialist tests for outpatient services. In that case, the operating profit would be reduced by €448 881 and amount to only €21 541, which combines both models of reducing the tests. If the threat of reduction in the number of tests performed was removed, continuous development and market surveillance are needed. Reducing expenses in all categories, which are greater in the developed European countries than in the United States (19), by carefully monitoring the labor process may lead to improved business performance of the clinical laboratory irrespective of the way in which it is financed. There is a possibility to realize various combinations of mentioned models, and their individual presentation enables computer modeling of economic operations.

According to the SWOT, profit and loss account, and economic sensitivity analyses, it is possible to quantify the effects of each described change. If all threats became a reality, their sum would amount to €448 881, and the sum of total opportunities only to €65.381. Therefore, total risk resulting from the threats is seven times greater than the possible growth based on the opportunities. In other words, the results showed that there is a possibility to reduce the operating profit from €470 723 to only €21 542, which is a 95% decrease, and the possibility to increase the operating profit to €535 804, which is a 14% increase. A new laboratory seems to be the greatest threat.

The financial situation in the health care system in the Republic of Croatia is unfavorable, but this type of analysis could lead to a positive financial shift despite the fact that making profit is not the primary function of the health system. Every health department manager should have basic knowledge of economic principles and their application. By using the knowledge of economy and analysis presented in this article, the Clinical Laboratory of Našice General County Hospital may increase its profitability and become a role model for other laboratories in business performance analysis.
